# The effect of garlic and stevia extract with aerobic exercise on hypothalamic leptin and ghrelin receptor mRNA expression and insulin resistance in obese rats

**DOI:** 10.1186/s12906-025-04756-7

**Published:** 2025-03-14

**Authors:** Zohre Amirkhani, Armin Morteza Gholi, Sara Asghari, Danial Hakak, Mahdi Pouryousef, Behrooz Yahyaei, Sayyed-Javad Ziaolhagh

**Affiliations:** 1 Department of Exercise Physiology, Mashhad Branch, Islamic Azad University, Mashhad, Iran; 2 Department of Medical Sciences, Shahrood Branch, Islamic Azad University, Shahrood, Iran; 3 Department of Exercise Physiology, Bojnourd Branch, Islamic Azad University, Bojnourd, Iran; 4 Department of Exercise Physiology, Neyshaboor Branch, Islamic Azad University, Neyshaboor, Iran; 5 Department of Exercise Physiology, Faculty of physical education and sport science, Kharazmi University, Tehran, Iran; 6 Department of Medical Sciences, Biological Nanoparticles in Medicine Research Center, Shahrood Branch, Islamic Azad University, Shahrood, Iran; 7 Department of Exercise Physiology, Shahrood Branch, Islamic Azad University, Shahrood, Iran

**Keywords:** Leptin receptor mRNA, Ghrelin receptor mRNA, Insulin resistance, Aerobic exercise, Obesity

## Abstract

**Background:**

Obesity has recently become the most prevalent diet-related disease worldwide. This study aimed to investigate the effects of aerobic exercise, stevia, and garlic extract on leptin and ghrelin receptor mRNA, as well as the interactions between insulin and glucose in relation to these genes.

**Methods:**

A total of 50 male Wistar rats were split into two groups: one group was fed regular rodent food, while the other was fed a high-fat diet for 12 weeks to induce obesity. The obese rats were then divided into six groups of five (*N* = 5) based on Lee’s index: an obese control group (OC), obese treated with garlic, stevia (OGS), obese aerobic exercise (OE), obese garlic and aerobic exercise (OGE), Obese stevia and aerobic exercise (OSE), and a Normal weight group (NC). The exercise groups participated in a gradually increasing aerobic walking program, whereas the stevia and garlic groups received daily oral doses of their respective extracts. The expression of leptin and ghrelin receptor genes in the hypothalamus was measured using real-time PCR, and insulin resistance was calculated using the HOMA/IR formula. Statistical analysis was conducted using ANOVA, with a significance level set at *P* < 0.05.

**Results:**

The obese (O) rats experienced a significant increase in body weight compared with the control (C) rats (*p* = 0.013), whereas the treated rats showed no significant changes in weight. Leptin receptor mRNA levels were significantly lower in O rats than in C rats (*p* = 0.00), but increased significantly in the OS (*p* = 0.000) and OSE (*p* = 0.034) groups compared with obese rats. Ghrelin receptor mRNA levels were significantly higher in the O group than in the C group (*p* = 0.035), but decreased in all treatment groups, with the OE (*p* = 0.001), OGE (*p* = 0.001), and OSE (*p* = 0.004) groups showing the greatest reductions. Insulin resistance increased slightly in the O group compared with the C group (*p* = 0.112), but was lowest in the OS group among all groups (*p* = 0.018).

**Conclusions:**

Stevia significantly improved leptin and ghrelin receptor mRNA expression, glucose levels, and insulin resistance in obese rats, showing its potential as an effective dietary intervention for managing obesity.

## Introduction

Obesity, a complex multifactorial disorder, has reached epidemic proportions worldwide and is associated with significant morbidity and mortality. Excessive accumulation of adipose tissue and dysregulation of energy homeostasis characterize this condition, resulting in an imbalance between energy intake and expenditure. Understanding the neuroendocrine mechanisms involved in appetite regulation and energy balance is crucial for developing effective strategies to combat obesity [1]. The hypothalamus, a key region in the brain, plays a vital role in regulating energy balance through the actions of various neuropeptides and hormones [2]. Leptin and ghrelin, two important hormones secreted by adipose tissue and the stomach, respectively, have emerged as key players in regulating appetite and maintaining energy balance. Leptin acts as a satiety hormone, suppressing appetite and promoting energy expenditure, whereas ghrelin is a hunger hormone, stimulating appetite and increasing food intake. Leptin and ghrelin exert their effects by binding to specific receptors in the hypothalamus, namely the leptin receptor (LepR) and the growth hormone secretagogue receptor (GHSR), respectively. These receptors are expressed in specific hypothalamic nuclei that are involved in regulating appetite, such as the arcuate nucleus and paraventricular nucleus (PVN) [3]. Previous studies have provided valuable insights into the role of leptin and ghrelin receptor mRNA in the hypothalamus and their involvement in the pathophysiology of obesity. For instance, research has shown that leptin resistance, which is characterized by reduced responsiveness to leptin, is a common feature of obesity. Impaired signaling through LepR in the hypothalamus leads to deregulated appetite control and disrupted energy balance [4]. Similarly, changes in ghrelin signaling and GHSR expression have been observed in obesity, leading to increased appetite and decreased energy expenditure [5]. Several studies have investigated the molecular mechanisms underlying leptin and ghrelin receptor signaling in the hypothalamus. For example, Borg et al. demonstrated that leptin activates proopiomelanocortin (POMC) neurons and inhibits neuropeptide Y/agouti-related peptide (NPY/AgRP) neurons in the ARC, leading to a decrease in appetite [6]. Moreover, Hansen et al. found that ghrelin promotes the SIRT1-p53 and CaMKK2-AMPK pathways in the ventromedial nucleus of the hypothalamus (VMH), thereby modulating appetite and energy balance [7]. Understanding the complex interaction between leptin, ghrelin, and their receptors in the hypothalamus is essential for uncovering the mechanisms that contribute to obesity and for developing specific therapeutic interventions. The effects of Allium sativum (garlic) and Stevia rebaudiana extracts on obese rats have been studied. A study on male Wistar rats fed with a high-fat diet found that solo garlic extract reduced low-density lipoprotein (LDL) and increased superoxide dismutase (SOD) levels, indicating potential benefits for blood lipid profiles and antioxidant activity [8]. Another study on black garlic (Allium sativum Linn) found that it significantly reduced the body weight of obese rats, but did not significantly reduce heart and aortic weight or histopathological features [9].In the case of Stevia rebaudiana, research has shown its antihypertensive, antihyperglycemic, and antioxidant effects on rats with induced metabolic syndrome [10]. Additionally, Stevia rebaudiana extracts have been found to attenuate metabolic dysfunctions in the skeletal muscles of type 1 diabetic rats via AMPK upregulation and antioxidant activities [11]. While these studies provide valuable insights into the effects of the individual extracts on rats, there is a lack of specific research on the modification of these extracts in obese rats. Therefore, the direct effects of modified garlic and Stevia rebaudiana extracts on obese rats remain to be investigated.

Aerobic exercise has long been recognized as a crucial component of weight management and the improvement of overall metabolic health. It plays a significant role in modulating energy balance, enhancing insulin sensitivity, and regulating appetite through its effects on the hypothalamus. Specifically, it affects the receptors for leptin and ghrelin, which are crucial regulators of appetite and energy expenditure [12]. Previous studies have examined the impact of aerobic exercise on leptin and ghrelin receptor mRNA levels in the hypothalamus, offering valuable information on the possible mechanisms that contribute to exercise-induced alterations in appetite control and energy equilibrium [6]. Exercise improves leptin sensitivity, reduces leptin resistance, and restores the ability of leptin to effectively suppress appetite. This effect may involve alterations in the expression and activity of leptin receptor mRNA in the hypothalamus, enabling improved signaling and enhanced appetite control. Similarly, aerobic exercise has been found to influence ghrelin signaling in the hypothalamus. Research suggests that exercise may decrease circulating ghrelin levels and modulate ghrelin receptor mRNA expression in the hypothalamus, potentially contributing to appetite suppression and reduced food intake [13].

Garlic (Allium sativum), a widely used culinary and medicinal herb, has garnered attention for its potential health benefits. These benefits include its effects on obesity and the regulation of appetite through the modulation of leptin receptor mRNA in the hypothalamus [14]. Several studies have explored the anti-obesity properties of garlic and its impact on the expression and activity of these receptors, providing insight into its potential therapeutic value [15]. In vitro, animal, and human studies have demonstrated that allicin, via the KLF15 signaling cascade, can prevent obesity and related metabolic disorders by increasing the expression of genes specific to brown adipocytes, such as UCP-1 [16].

Stevia, a commonly used natural sweetener, stimulates insulin production by inhibiting ATP-dependent potassium channels and suppressing glucagon secretion by pancreatic alpha cells. It does not achieve this by inducing incretin hormones [17]. Stevia also has regulatory effects on pancreatic beta cells, enhancing insulin release in response to glucose by opening calcium channels. Although nonnutritive sweeteners were traditionally considered metabolically inert, emerging research suggests that they may have physiological effects on glucose metabolism and appetite stimulation. Much of this research revolves around the discovery of sweet taste receptors T1r2 and T1r3 in the oropharynx, as well as in enter endocrine cells of the intestine and pancreas, which stimulate the secretion of glucose-insulin tropic peptide (GIP) [18]. Therefore, this study aimed to investigate the potential anti-obesity effects of garlic and stevia extracts along with aerobic exercise on hypothalamic leptin and ghrelin receptor mRNA levels, as well as insulin resistance, in obese male Wistar rats.

## Methods

### Study design

**Fig. 1:**
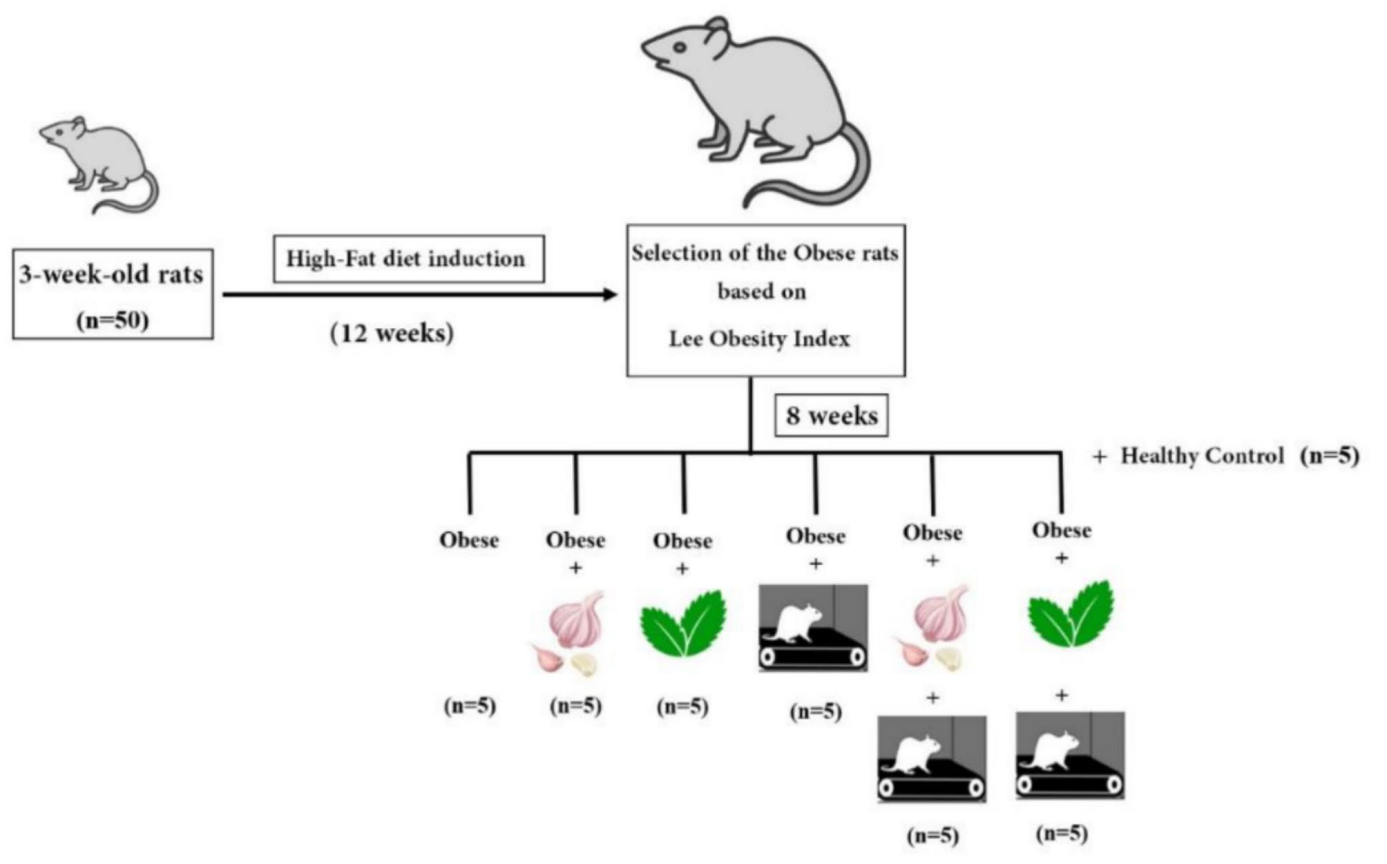
The figure depicts the study design used in the research, highlighting the induction of obesity, subject selection, and extract and exercise treatment groups

*This research protocol was approved by the Research Ethics Committees of Shahrood Islamic Azad University.* At 3 weeks of age (after the end of infancy), 50 male Wistar rats were purchased from the Animal Services laboratory and kept at the University’s Physiology Laboratory on an 8: 16 h light/dark cycle (lights on 07.00 A.M). The rats were housed in standard cages (We used standard rat cages with dimensions of 18 inches (45 cm) in width, 12 inches (30 cm) in height, and 18 inches (45 cm) in length. These provided ample space for the rats to move and exhibit natural behaviors), and had free access to water; however, they were given conventional rat chow for the control group and a high-fat diet [19] for the obesity-induced groups for 12 weeks. The obese rats were then divided into six groups of five (*N* = 5) based on Lee’s index [20]: an obese control group (OC), obese treated with garlic (OG), stevia (OS), obese aerobic exercise (OE), obese garlic and aerobic exercise (OGE), Obese stevia and aerobic exercise (OSE), and a Normal weight group (NC) (Fig. 1).

### Exercise protocol

Aerobic exercise was performed on a rodent treadmill (5 Lanes, Daneshsalar Iranian Co.). According to the exercise performance capacity of rats with induced obesity, the exercise intensity was varied based on the program proposed by Kim et al. [21] and Choi et al. [22] and was practiced for 8 weeks. Table 1 shows the exercise protocol.

**Table 1:** Modified aerobic Exercise protocol in rodents Treadmill. The table displays the duration, speed, inclination, and frequency of aerobic exercise sessions for each group

Speed (m/min)	Time (min)	Grade (%)	Frequency (day/week)
**8**	5	0	5
**11**	5		
**15**	20		
**8**	10		

### Extract preparation and administration

#### Garlic allium sativum

To produce the garlic extract, fresh garlic was first prepared at the Daru Essans Company in Gorgan City and processed into garlic extract. The garlic was cleaned, minced, and scoured in 96% ethanol for 48 h and then centrifuged at 200 g for 5 min. The supernatant was then filtered before being evaporated at 40 °C, and the extract was stored frozen at -20 °C. At the time of extract use, the required amounts were combined with normal saline (0.9% NaCL) and added to the daily water consumption (50 ml) to the extent of 250 mg/kg [23].

#### Stevia rebaudiana

Fresh stevia was purchased from Gorgan, Iran, and 50 g of dry powder was macerated with 100 ml of 70% (w/v) ethyl alcohol in a round bottom flask for a week with occasional stirring, and stevia extract was obtained. The extract was then filtered for gross residues using a muslin cloth and then through Whatman filter paper before being stored at 4 °C for later use. Stock solutions of crude extracts were prepared by mixing sufficient dry extracts with the inert solvent dimethyl sulfoxide to obtain final concentrations. Stevia extract was also added to the daily water intake at a concentration of 250 mg/kg [24].

### Specific laboratory determination

*Animals were starved overnight (12 h) with unrestricted access to water after the trial and then anesthetized with intraperitoneal ketamine (60 mg/kg body wt.) -Xylazine (5 mg/kg body wt.) between 09:00 and 11:00 AM. Thirty minutes later*,* blood was drawn by cardiac puncture*,* and serum was separated and stored at -20 degrees Celsius. After inducing anesthesia and performing direct blood collection from the heart*,* and then separating the heart (for euthanasia)*,* the animal’s skull was dissected to access the brain regions.* The brain was taken, and the hypothalamus was dissected using markers of the optic chiasma, lateral nuclei, mammillary bodies, and a depth of 2 mm after the brain was removed. The tissue samples were snap-frozen in liquid nitrogen and maintained at a temperature of -70 degrees Celsius in an ultra-low-temperature freezer. The glucose oxidase method with an analytical range of 0–700 mg/dl was used to measure plasma glucose on a Synchron CX9chemistry analyzer (Beckman Coulter, USA) using manufacturer-supplied kits. Each batch of plasma glucose was subjected to three levels of internal quality control (low, normal, and high). Microparticle enzyme immunoassay was used to measure serum insulin levels using an Abbott Axsym Immunoassay Analyzer (Abbot Diagnostics, USA) and manufacturer-supplied kits. Each batch of serum insulin was subjected to three levels of internal quality control (low, normal, and high). The laboratory used Bio-(BioRad Rad’s Laboratories, US) external quality assurance services for plasma fasting glucose and serum insulin. The kits used for determining glucose and insulin concentrations were sourced from Pars Azmoon- IRAN. These kits were selected due to their reliability and accuracy, ensuring precise measurements throughout our experiments. The following formula was used to calculate insulin resistance using HOMA-IR [25]: HOMA-IR = [Fasting glucose (mg/dl] × Fasting Insulin (µU/ml]) ÷ 405.

### RT-PCR analysis of leptin and ghrelin mRNA in the hypothalamus

To specifically analyze leptin and ghrelin mRNA in the hypothalamus using RT-PCR, first isolate RNA from hypothalamic tissue samples using an RNA extraction kit. Synthesize cDNA from the RNA using reverse transcription with specific primers. Prepare a PCR mix with the cDNA, leptin and ghrelin primers, Taq polymerase, and dNTPs. Run PCR with denaturation at 95 °C for 5 min, followed by 30–35 cycles of denaturation at 95 °C for 30 s, annealing at 55–60 °C for 30 s, and extension at 72 °C for 1 min, ending with a final extension at 72 °C for 10 min. Separate the PCR products on a 1–2% agarose gel, stain with ethidium bromide, and visualize under UV light to confirm the presence of leptin and ghrelin mRNA, including appropriate positive and negative controls for validation. Detailed information on the primers can be found in Table 2.

**Table 2:** Gene’s primers. The table presents primer information for the targeted genes, including gene names, forward and reverse primer sequences, and the resulting amplicon size in base pairs

Name	Primer sequences (5^’^-3^’^)	Product size (bp)
*Leptin receptor*	*F: CTGGGTTTGCGTATGGAAGT* *R: CCAGTCTCTTGCTCCTCACC*	***217***
*Ghrelin receptor*	*F: CTGGACAAAGTCGAGCATCA* *R: CTGCCCATCTGGCTCTACTC*	***179***
*GAPDH* *(Housekeeping)*	*F: GTCTCCTCTGACTTCAACAGCG* *R: ACCACCCTGTTGCTGTAGCCAA*	***183***

### Statistical analysis

The fold change in gene expression was calculated as the ratio of the target gene’s expression level normalized to the housekeeping gene (GAPDH). The mean standard error (SE) of the fold change differences between groups was then computed [26]. Statistical analysis was performed using SPSS 26.0 and Prism 8 softwares. The Kolmogorov-Smirnov test assessed the normality of the data; if the distribution was not normal, a nonparametric test such as the Kruskal-Wallis test was used. Levene’s test evaluated the equality of variances. For normally distributed data with equal variances, one-way ANOVA followed by the Least Significant Difference (LSD) post hoc test was conducted to analyze group differences, with *p* < 0.05 considered statistically significant.

## Result

**Fig. 2:**
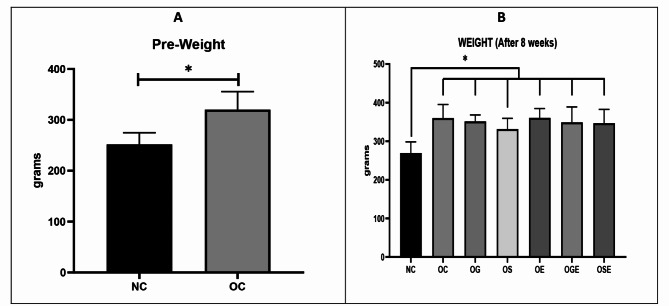
Weight differences after 12 weeks of high-fat diet (**A**) and after 8 weeks of treatment (**B**). Statistical analysis was performed, and a p-value of 0.05 was considered statistically significant, denoted by the symbol “*”

When compared with the control group, the induction of a high-fat diet for 12 weeks resulted in a significant increase in the body weight of juvenile rats (*p* = 0.013). Throughout the subsequent 8-week treatment period involving extract administration and training, the body weight of the rats remained elevated (Fig. 2A). Although the group receiving stevia extract exhibited a lower mean weight compared with the other groups, no statistically significant differences were observed between obese rats in the various treatment groups (*p* = 0.112) (Fig. 2B).

**Fig. 3:**
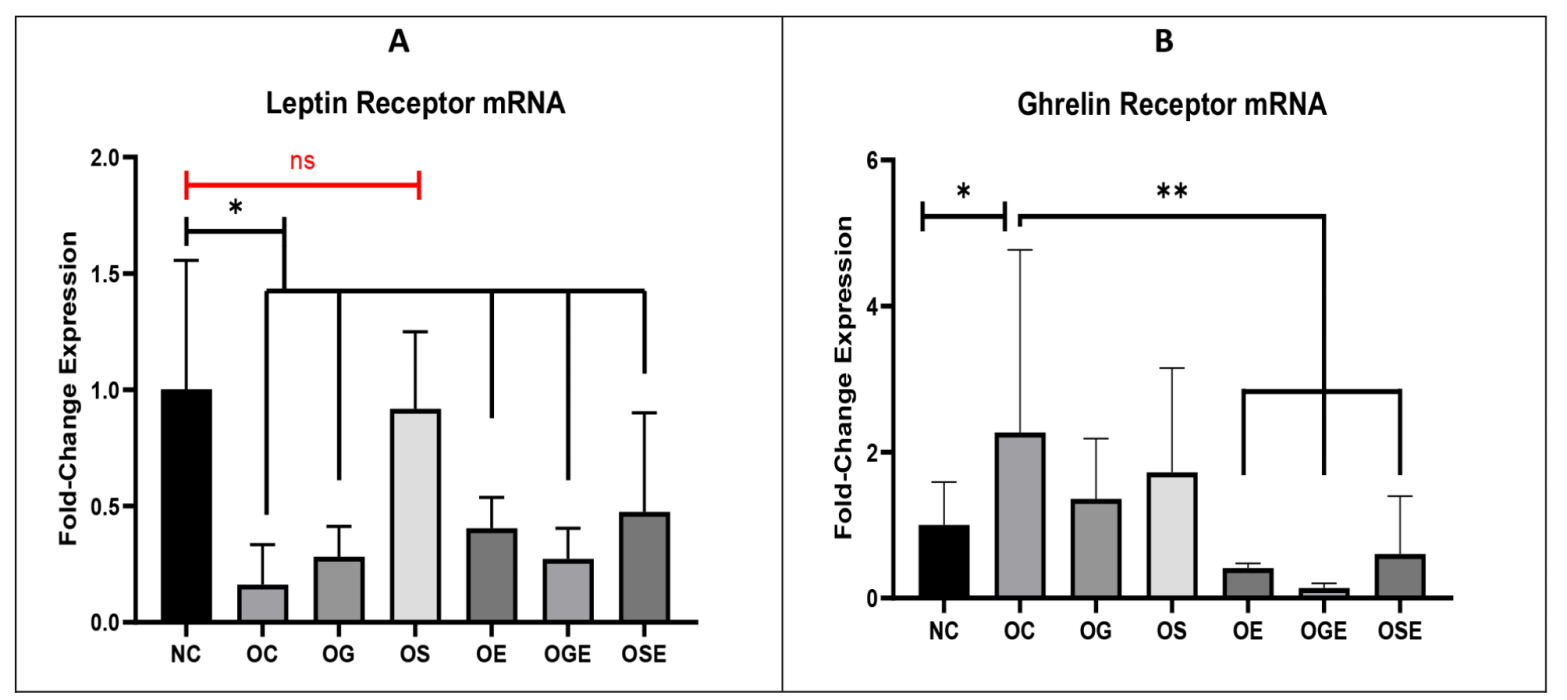
Fold change differences in leptin (**A**) and Ghrelin (**B**) receptor mRNA between the respective groups. Statistical analysis was performed, and a p-value of 0.05 was considered statistically significant, denoted by the symbol “*, **”. Ns: None Significant

Expression of leptin and ghrelin receptor mRNA was observed in the hypothalamus of both obese and control rats (Fig. 3C and D). In obese rats, a significant downregulation of leptin receptor mRNA was observed compared with the healthy control group (*p* = 0.001). Treatment with stevia extract (*p* = 0.001) and stevia extract combined with aerobic exercise (*p* = 0.034) significantly increased the number of leptin receptor mRNA in the hypothalamus compared with that in obese rats (Fig. 3C). In addition, ghrelin receptor mRNA increased significantly in obese rats compared with the control group (*p* = 0.035). In the treatment groups, rats in all exercise groups, such as aerobic exercise (*p* = 0.001), receiving garlic extract in conjunction with aerobic exercise (*p* = 0.001), and receiving stevia extract along with aerobic exercise (*p* = 0.004) demonstrated reduced levels of ghrelin receptor mRNA expression in their hypothalamus (Fig. 3D).

**Fig. 4:**
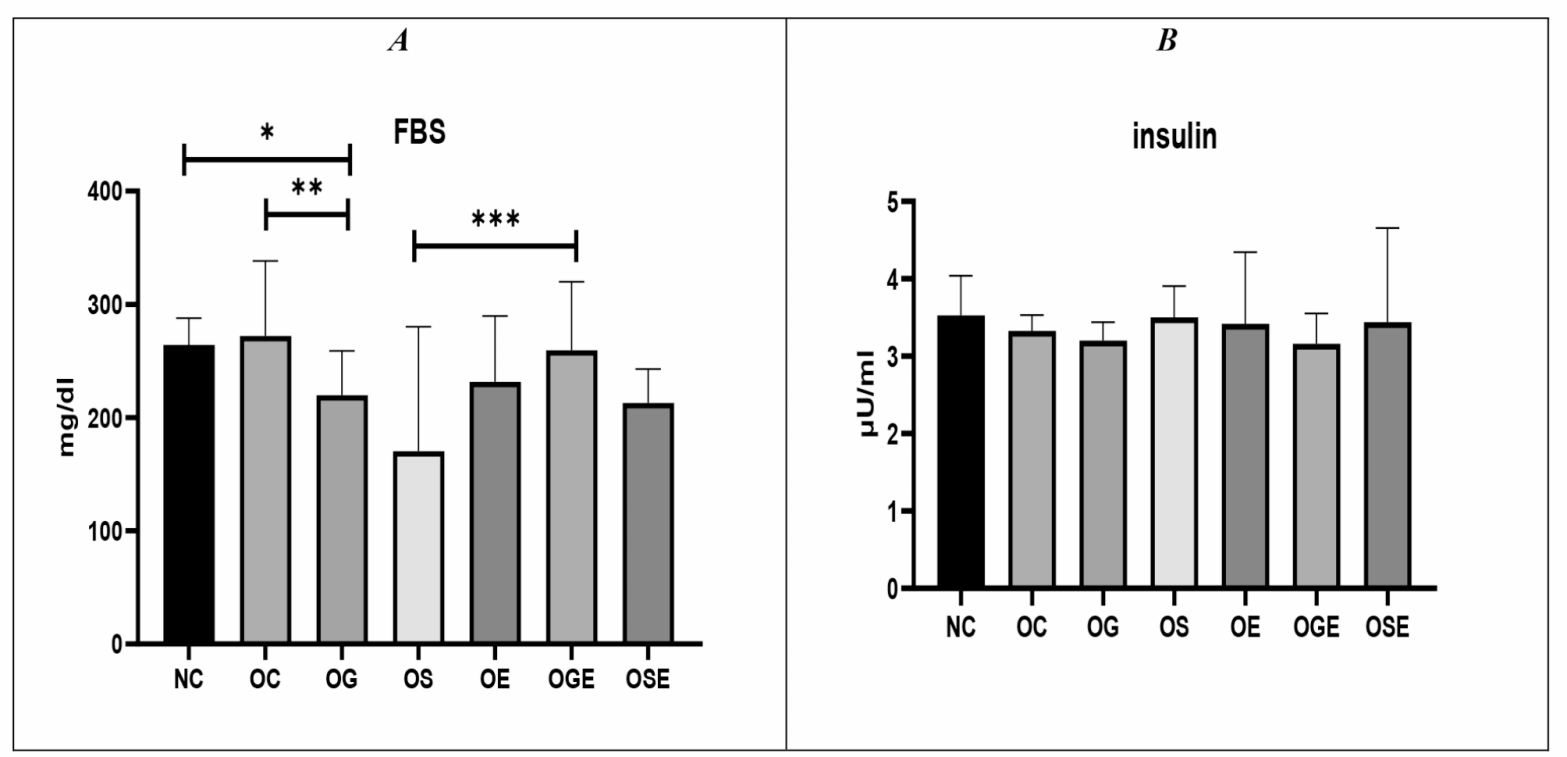
Serum levels of glucose (**A**) and insulin (**B**) measured in the different groups. The groups represent distinct experimental conditions or treatment interventions. Serum levels of glucose and insulin were quantified and compared between the groups. Statistical analysis was conducted, and a p-value of 0.05 indicates statistical significance, represented by the symbol “*, ** and ***”

**Fig. 5:**
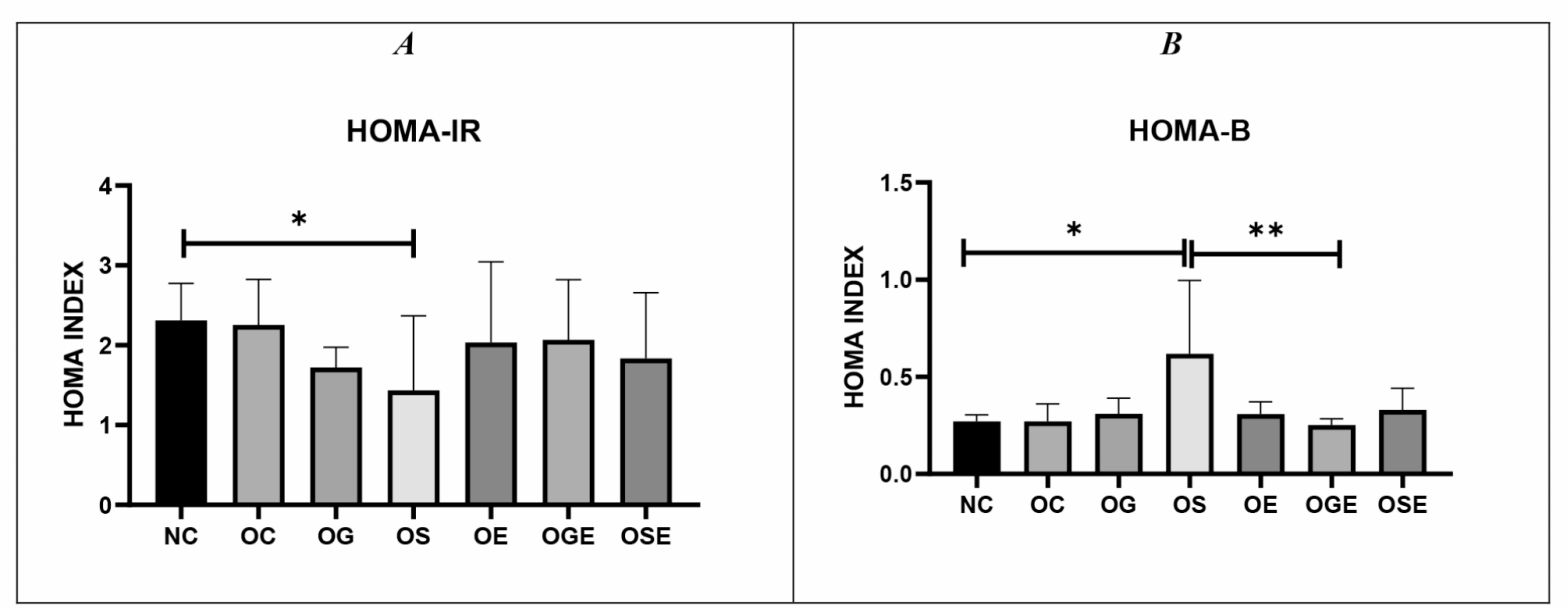
Comparison of insulin resistance calculated by HOMA-IR (**A**) and Pancreatic function calculated by HOMA-B (**B**) between groups. Statistical analysis of the data revealed a significant difference between the groups, with a p-value of 0.05 denoted by the symbol “*,**”

Although the fasting blood glucose levels of obese rats did not significantly differ from those of healthy control rats (*p* = 0.796), rats administered stevia showed significantly lower fasting blood glucose levels (*p* = 0.003). Additionally, the results revealed that obese rats had higher glucose levels compared with both the stevia with aerobic activity group (*p* = 0.041) and the stevia alone group (*p* = 0.001) (refer to Fig. 4A). Moreover, insulin levels in the obese treatment groups were insignificantly lower than those in the healthy control group (*p* = 0.559) (refer to Fig. 4B). Furthermore, while insulin resistance displayed a non-significant increase in obese rats compared with the healthy control group (*p* = 0.112), obese rats treated with stevia exhibited the lowest level of insulin resistance among all the groups (*p* = 0.018) (Fig. 5A). Inanition, pancreatic function calculated by HOMA-B formula showed that in stevia receiving group the change was significant compared to healthy control group (*p* = 0.0002) (Fig. 5B).

## Discussion

This study aimed to investigate the effects of an 8-week regimen of garlic and stevia extract along with aerobic exercise on the expression of hypothalamic leptin and ghrelin receptor genes as well as insulin resistance and weight gain in obese rats. Although a 12-week induction of high-fat diets resulted in a significant increase in the weight of the obese rats, the weight of the rats did not change appreciably in any group (Fig. 2). Leptin receptor mRNA levels were decreased in obese rats compared with the control group, but significantly increased in the stevia group (almost double that in the control group) and the stevia + aerobic activity group (Fig. 3). Ghrelin receptor mRNA in obese rats was much higher than that in the control group but was significantly lower (even lower than in control rats) in the stevia, stevia + exercise, and garlic + exercise groups (Fig. 4). The stevia and stevia + aerobic activity groups had significantly lower glucose levels than healthy control rats, but insulin levels did not change in either group (Fig. 5A). Although insulin levels were similar in all groups, insulin resistance was significantly reduced in the Stevia group (Fig. 5B).

The observed weight difference in our study underscores the impact of a high-fat diet on juvenile rats. Over the 12-week induction period, rats on the high-fat diet exhibited a significant increase in body weight compared to the control group (*p* = 0.013). Despite the implementation of various treatments during the subsequent 8-week period, including garlic and stevia extract administration and aerobic exercise, the elevated body weight was sustained. Notably, the stevia extract group showed a lower mean weight relative to other groups, though this was not statistically significant (*p* = 0.112). These findings suggest that while dietary interventions like stevia may offer some benefits in weight management, the challenges of reducing weight post-obesity onset remain substantial. Further research is required to elucidate the mechanisms underlying these effects and to optimize treatment strategies for obesity management.

Consistent with our results, many studies have shown that physical activity induces many adaptations in the central nervous system. This treatment enhanced the thickness and integrity of the basal lamina and blood-brain barrier, reduced oxidative stress caused by mitochondria in neurons and activated leukocytes, and increased the activity of antioxidant enzymes. As a result, there was an upregulation of integrin expression, which plays a crucial role in connecting endothelial cells to astrocytes. Additionally, the treatment lowered levels of matrix metalloproteinase-9, an enzyme produced by endothelial cells, microglia, and astrocytes that primarily degrades the extracellular matrix and influences basal lamina growth, among other metabolic and physiological effects [27]. In response to exercise, peripheral organs, including adipose tissue, skeletal muscle, and liver, can release numerous hormones that are beneficial to brain and metabolic health. Animal studies have shown that obesity inhibits cell proliferation, neuronal differentiation, and cell survival in newborn dentate Gyrus cells in the hippocampus [28]. The impact of physical activity on leptin levels is controversial. Previous studies have suggested that exercise may lower leptin levels or have no effect at all. As mentioned above, there is a positive relationship between leptin levels and weight. Therefore, weight loss through exercise can lead to lower leptin levels. However, there is conflicting evidence that other modulators of the leptin system, such as cortisol, upregulate leptin in response to stimuli such as exercise [29]. To date, few studies have been conducted directly on leptin and ghrelin receptor mRNA in the hypothalamus. The question of whether exercise alters appetite, regardless of its long-term effects on energy balance, has long been studied. In both lean and obese animals, hypothalamic leptin sensitivity improves immediately after intense exercise. According to our findings, exercise combined with stevia increased leptin receptor mRNA and decreased ghrelin receptor mRNA in the hypothalamus of obese rats. Exercise appears to affect these receptors by activating intracellular calcium and catecholamines, thereby increasing adipocyte and fatty acid metabolism. Exercise also appears to suppress mTOR, thereby increasing insulin sensitivity, fat breakdown, and leptin production and signaling [29].

Leptin resistance is a typical symptom of a high-fat diet in mice and is caused by decreased leptin transport across the blood– brain barrier and decreased activation of leptin receptor signaling. A high-energy diet followed by increasing leptin and insulin levels reduced leptin mRNA in the hypothalamus of diet-obese rats, and leptin mRNA was further reduced in the ventromedial hypothalamic nuclei [4]. Our results showed that in the hypothalamus, the expression of leptin receptor mRNA was significantly lower in the obese group than in the control group. In the current study, a direct comparison of sedentary rats fed low-fat and high-fat diets revealed the development of significant leptin resistance in obese rats (Fig. 3A). In addition, our results showed that stevia can dramatically increase leptin receptor mRNA (Fig. 3B) in rats receiving it, which is believed to be involved in glucose metabolism and insulin production. Stevia can affect glucose metabolism in two ways. First, stevia contains several glycosides that cannot be broken down or absorbed by the digestive tract. Therefore, consuming stevia sweeteners does not affect blood sugar levels. Our results are consistent with those of several studies that have found significant reductions in blood glucose levels after stevia supplementation [30] (Fig. 5A). Stevia’s second regulatory role is to promote insulin release by activating calcium channels in pancreatic beta cells in response to glucose [31]. Lower insulin resistance in rats fed Stevia appears to be the main cause of changes in leptin and ghrelin receptor mRNA levels in this study. Lower insulin resistance has been associated with improved leptin sensitivity in previous studies [32]. Insulin resistance, characterized by reduced responsiveness to insulin, can interfere with the normal signaling pathways of various hormones, including leptin. When insulin resistance is present, it can disrupt the communication between insulin and leptin receptors, leading to impaired leptin signaling and reduced sensitivity to its effects on appetite regulation and energy balance. Conversely, decreased insulin resistance, as observed in the stevia-fed group, may contribute to enhanced leptin sensitivity, allowing for more effective regulation of hunger and satiety cues. Existing research shows that stevia, garlic, and aerobics affect the complex functions of glucose and insulin at leptin and ghrelin receptor mRNA levels. Insulin has a dual effect in regulating leptin: an early inhibitory effect (less than 48 h) followed by stimulation (48 to 96 h later). The inhibitory phase was observed at all glucose concentrations studied (ranging from 1 to 25 mM), but the stimulatory phase required physiological or supraphysiological glucose concentrations [33]. In addition, it was shown that ghrelin receptor mRNA levels were lower in the high-fat rats than in the control group. According to a recent study, increased plasma levels of ghrelin and preproghrelin mRNA expression during stress may help reduce the symptoms of stress-related sadness and anxiety. Furthermore, an association between short sleep duration and high levels of ghrelin, as well as obesity, was found when decreasing ghrelin concentrations were found with increasing sleep duration [34]. The data reported in the signaling section also showed that ghrelin mRNA, gastric pre-protein, and circulating ghrelin are affected by the activation of gastric insulin signaling. Results from two separate studies in mice also suggest that a hyperinsulinemic state might increase ghrelin mRNA expression, although no information on protein levels has been provided [35]. The obese rats had slightly lower insulin levels than the control group (Fig. 5B), which is consistent with other insulin-related studies. Further investigation is needed to determine the cause of ghrelin-induced fluctuations in insulin secretion and glucose homeostasis. Stevia extract stimulates insulin production by inhibiting ATP-dependent potassium channels and reducing glucagon secretion by pancreatic alpha cells instead of stimulating incretin hormones. Stevia also plays a regulatory role in pancreatic beta cells, where it opens calcium channels that trigger insulin release in response to glucose [36]. It has also been found that stevia can prevent obesity by reducing calorie consumption. However, one study found that administering stevia at doses approximately 100 times higher than the Acceptable Daily Intake (ADI) led to a reduction in food intake and body weight in adult female rats after 12 weeks of regular treatment. Recent research suggests that stevia may have physiological effects on glucose metabolism and appetite stimulation [37]. Moreover, garlic or its sulfur-containing components have anti-obesity properties in in-vitro, animal, and human studies. In addition, allicin potentially prevents obesity and metabolic diseases associated with increased expression of brown fat-specific genes [16]. In diet-induced obese rats, oral consumption of fermented garlic extract for 8 weeks reduces body weight, fat burning, triglyceride, and total cholesterol levels and suppresses adipogenesis [38]. In obese rats treated with garlic oil, body weight decreased and liver damage was prevented. Studies have shown that raw garlic is effective in improving insulin sensitivity and reducing metabolic syndrome and oxidative stress in rats. In addition, the effect of garlic on glucose and insulin and subsequently its effect on brain insulin receptors and cascades involved in appetite have been reported [39]. Numerous studies have been conducted on the effects of garlic (in various forms of supplementation) and its derivatives in various animal models of obesity. Garlic-derived compounds reduced adipocyte growth in vitro by activating AMPK, inhibiting acetyl-CoA carboxylase-1 (ACC-1), upregulating carnitine palmitoyltransferase (CPT-1), or activating ERK. Allicin has also been identified as a potential preventive agent for obesity and related metabolic diseases by increasing the expression of brown adipocyte-specific genes such as UCP-1 through the KLF15 signaling cascade [40]. Our results showed that garlic extract combined with aerobic exercise reduced ghrelin receptor mRNA levels, which may be due to garlic extract’s anti-obesity properties.

The significant increase in pancreatic function, as calculated by the HOMA-B formula, in the stevia-treated group compared to the healthy control group (*p* = 0.0002) underscores the potent effect of stevia on beta-cell function. This improvement suggests that stevia may enhance insulin secretion and beta-cell responsiveness, which are critical for maintaining glucose homeostasis. The marked elevation in HOMA-B index highlights stevia’s potential as a dietary supplement in improving pancreatic health and combating insulin resistance. These findings align with previous studies indicating that natural extracts can positively modulate metabolic pathways, presenting stevia as a viable intervention for managing obesity and its metabolic complications. Future research should explore the molecular mechanisms through which stevia exerts these effects, providing deeper insight into its therapeutic potential.

## Conclusions

In conclusion, our study demonstrated that 12 weeks of a high-fat diet significantly increased weight and ghrelin receptor mRNA levels, while decreasing leptin receptor mRNA, without notably affecting glucose or insulin levels. However, after 8 weeks of treatment with stevia, garlic, and aerobic exercise, the stevia group exhibited the most significant changes in leptin and ghrelin receptor mRNA expression, glucose levels, and insulin resistance. These findings highlight the potential of stevia as a dietary supplement for managing obesity and its associated metabolic disorders. Further research is essential to confirm these results and elucidate the underlying mechanisms. Overall, our study offers valuable insights into the benefits of integrating dietary interventions with exercise for obesity treatment.

## Data Availability

All data generated or analysed during this study are included in this published article.
